# Hemophagocytic Lymphohistiocytosis as First Manifestation of Dual B‐Cell Neoplasms: A Case Report of Co‐Existing Multiple Myeloma and B‐Cell Lymphoma

**DOI:** 10.1002/jha2.70210

**Published:** 2026-01-22

**Authors:** Carla Romagnoli, Alexandra Lyubimova, Leily Santos‐Carrion, Ferial Alloush, Vathany Sriganeshan, Andrea Noboa, Jacqueline C. Barrientos

**Affiliations:** ^1^ Mount Sinai Comprehensive Cancer Center Miami Beach, Florida USA; ^2^ Sociedad de Lucha contra el Cáncer Guayaquil Ecuador

**Keywords:** hemophagocytic lymphohistiocytosis, hyperinflammation, lymphoma, plasma cell neoplasm, tocilizumab

## Abstract

Hemophagocytic lymphohistiocytosis (HLH) is an immune disorder causing excessive inflammation and tissue damage. Lymphoma, especially T‐cell lymphoma, is the most common cause of HLH, while Multiple Myeloma (MM) is rarely associated. We present a 61‐year‐old man with spiking fevers, fatigue, and unintentional weight loss. The HLH was driven by two distinct malignant clones in his bone marrow: plasma cell neoplasm and B‐cell lymphoma. Patient failed initial therapy with steroids, anakinra, and chemotherapy. Tocilizumab use, on the other hand, led to an excellent clinical and laboratory response immediately. This is the first reported case of HLH associated with two hematological malignancies.

**Trial Registration**: The authors have confirmed clinical trial registration is not needed for this submission

## 1 Introduction

Hemophagocytic lymphohistiocytosis (HLH) is an immune response syndrome marked by aberrant hyperinflammation, hyperferritinemia, and a potentially fatal cytokine storm, marked by persistent activation of CD8+ cytotoxic T lymphocytes and natural killer (NK) cells, leading to increased secretion of inflammatory cytokines and macrophage activation resulting in excessive inflammation and tissue destruction [1, 2].

It can be divided into two main forms: primary or hereditary form (usually a pediatric disease associated with autosomal recessive inheritance, caused by loss‐of‐function mutations in genes involved in the cytotoxic function of NK cells and CD8+T cells) and secondary forms (commonly associated with conditions with immune dysregulation, such as infection, malignancy, autoimmune disorders, or drug‐induced).

Malignancy‐associated HLH may occur as a result of malignancy itself or the therapeutic intervention. Lymphoma is the most common neoplastic cause, with T‐cell lymphomas being more frequent than B‐cell lymphomas, whereas only 0.24% of cases are associated with multiple myeloma (MM) [3, 4, 5]. HLH diagnosis requires five out of nine diagnostic criteria (“HScore”): fever; splenomegaly; cytopenias affecting two or three lineages (hemoglobin < 9 g/dL, platelets < 100,000/µL, and neutrophils < 1000/µL); hypertriglyceridemia and/or hypofibrinogenemia; hemophagocytosis in the bone marrow, spleen, lymph nodes, or liver; hyperferritinemia; low or absent NK cell activity; and increased soluble CD25 (IL‐2r) concentration (> 2400 U/mL) [3, 6].

HLH is extremely rare (reported at 1 per 800,000 people and 1–10 per 1,000,000 children). The mortality of secondary HLH is high, but statistics are limited by the small number of reported cases (Table 1). Case series of adults treated with a variety of regimens report a 30‐day mortality of 20%–44% despite early detection and appropriate therapy. Malignancy‐associated HLH portends the worst prognosis among all HLH subtypes with a five‐year overall survival ranging from 10%–30% [7].

**TABLE 1:** Characteristics of case reports of myeloma‐associated hemophagocytic lymphohistiocytosis (HLH).

Age (in years)/sex	Type of MM	HLH criteria	Possible triggers	HLH‐directed therapy	Chemotherapy	Outcome	Reference
59/M	IgA Lambda	Fever Hemophagocytosis Hyperferritinemia Increased sCD25 Hypertriglyceridemia	POD	no	DVR‐PACE	Bad response, switch to DP. Died after 5 months from POD	[6]
56/M	IgG Lambda	Fever Bicytopenia Hyperferritinemia Hemophagocytosis Increased sCD25	Not given	Dexamethasone and etoposide	No	Multiorgan failure after 8 weeks of treatment	[8]
29/not given	IgG Kappa	Fever Pancytopenia Hypofibrinogenemia Hyperferritinemia Hemophagocytosis	Lenalidomide related MM progression	Dexamethasone and etoposide	Withhold lenalidomide KD‐PACE Allo‐HSCT	HLH resolved shortly with quick POD	[9]
64/M	IgA Kappa	Fever Pancytopenia Splenomegaly Hyperferritinemia Hemophagocytosis	Disease	Methylprednisolone	Cyclophosphamide and dexamethasone	Multiorgan failure. Died after 15 days of hospitalization	[2]
59/M	IgG Lambda	Fever Pancytopenia Hyperferritinemia Increased sCD25	Respiratory syncytial virus Incomplete immune reconstitution after ASCT	Dexamethasone, tocilizumab and anakinra	Carfilzomib and daratumumab	On Day 4 of anakinra patient developed septic shock and passed away	[10]
78/M	IgA Kappa	Fever Bicytopenia Hyperferritinemia Hypofibrinogenemia Hemophagocytosis Low NK cell activity Increased sCD25	EBV infection. disease	Dexamethasone and anakinra	Cyclophosphamide, bortezomib and dexamethasone	Six months later VGPR. Normalized blood counts	[1]

Abbreviations: M, male; POD, progression of disease; DVR‐PACE, dexamethasone, bortezomib, lenalidomide, cisplatin, doxorubicin, cyclophosphamide, etoposide; DP, daratumumab/pomalidomide; KD‐PACE, carfilzomib, dexamethasone, thalidomide, cisplatin, doxorubicin, cyclophosphamide and etoposide; Allo‐HSCT, allogeneic hematopoietic stem cell transplantation; ASCT, autologous stem cell transplant; VGPR, very good partial response.

## 2 Case Report

A 61‐year‐old Ecuadorean man began experiencing fever and chills requiring hospitalization. Imaging of the chest, abdomen, and pelvis was unremarkable. A bronchoalveolar lavage demonstrated gram‐positive cocci and was treated with antibiotics, but he continued to spike fevers. Over the next 2 months, he lost 25 pounds and became fatigued. Infective endocarditis was ruled out, as well as COVID‐19 and dengue infections. His cell lines showed bicytopenia requiring blood transfusions. Because he had hypertriglyceridemia and hyperferritinemia, HLH was suspected, and he was started on high‐dose steroids while extensive workup was being done.

### 2.1 Clinical Findings

Initial laboratory examination at our center revealed anemia and thrombocytopenia in the setting of hyperferritinemia, hypertriglyceridemia, normal calcium, and elevated IL‐6 and IL‐2 soluble (see Table 2). Initial bone marrow biopsy performed in Ecuador showed trilineage hematopoiesis and megakaryocytic hyperplasia. We repeated a biopsy locally, which showed hemophagocytosis in the setting of myeloma (20%–30% of plasma cells by immunohistochemistry) and B‐cell lymphoma (Table 3 and Figures 4, 5, 6, 7, 8, 9, 10, 11). Along with clonal plasma cells in the bone marrow, the patient also exhibited anemia, meeting one of the CRAB criteria for MM.

**TABLE 2:** Patient characteristics and clinical data upon presentation.

Age	61
Gender	Male
Ethnicity	Hispanic/Latino
Symptoms experienced	Fever, unintentional weight loss, fatigue
HIV status	Negative
EBV (PCR)	Detected
Hgb	7.2 (14–18)
Neutrophils count	3.3 (1.8–7.2)
Platelet count	2400 (150–450)
Calcium	8.9 (8.5–10.1)
Triglycerides	405 mg/dL (30–150)
IL‐6 levels	116 pg/mL (< 5.00)
CD25s	21,875 pg/mL (532–1,891)
Ferritin	14,238 ng/mL (26–388)
Beta‐2‐microglobulin	4.010 mg/dL (1.09–2.53)
LDH	1,375 U/L (81–246)
Creatinine	1.10 mg/dl (0.7–1.3)
Kappa/Lambda ratio	0.17 (0.26–1.65)
Immunofixation	IgG lambda monoclonal protein, 0.96 g/dL
PET‐CT scan	No hypermetabolic adenopathy or bone lesions. Splenomegaly 13.9 cm

**TABLE 3:** Bone marrow characteristics.

	1st Biopsy (February 14, 2025)	2nd Biopsy (March 5, 2025)
Pathology	Hypercellular bone marrow for age with trilineage hematopoiesis and megakaryocytic hyperplasia (Figures 4 and 5).	Hemophagocytosis, along with a hypercellular bone marrow demonstrating 20%–30% of plasma cells (Figures 6, 7, 8, 9).
Flow cytometry	−	Two clones: ‐IgG Lambda monoclonal plasma cells ‐small subset of CD10+ kappa‐restricted monoclonal B cells. (Figures 10 and 11)
Karyotype	−	46 XY (20)
FISH analysis	−	Presence of t(11;14) (Plasma cell enrichment was performed using CD138 coated beads) NEGATIVE for 13q deletion, 1p32/1q21, p53, t(4;14), t(14;16), and t(14;20).
Molecular assay	−	MYD88 negative

**FIGURE 1:**
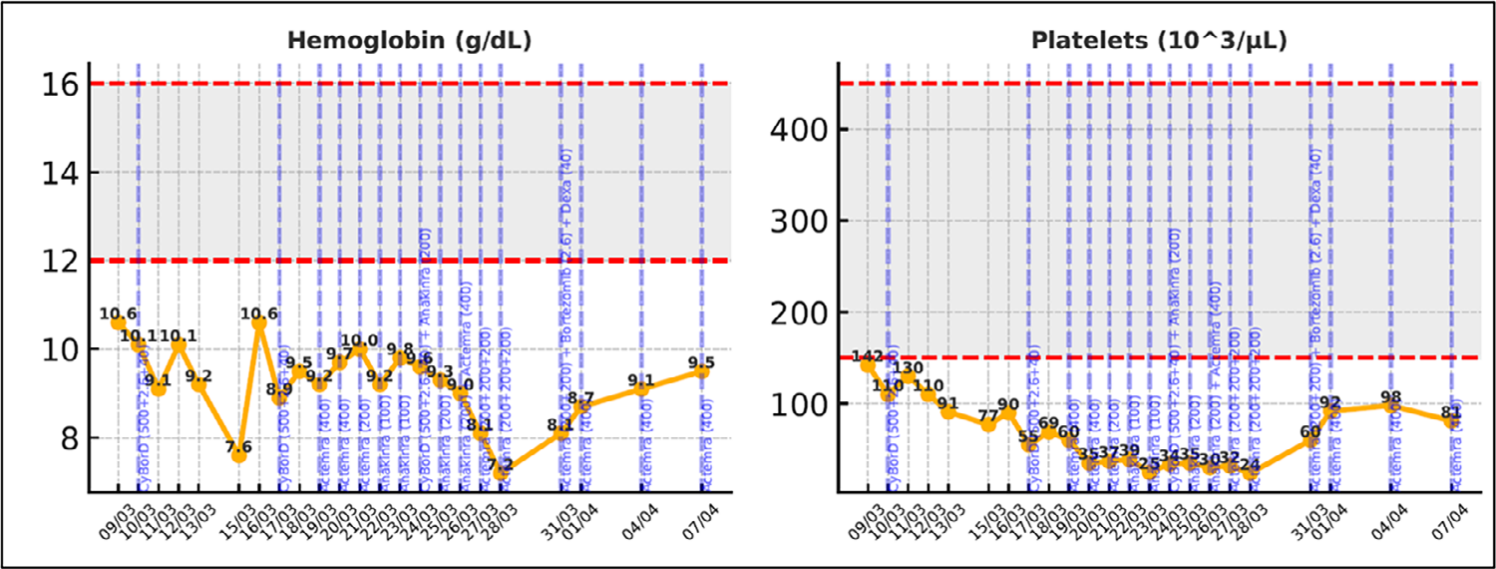
Evolution of Hgb and platelets following initiation of treatment.

**FIGURE 2:**
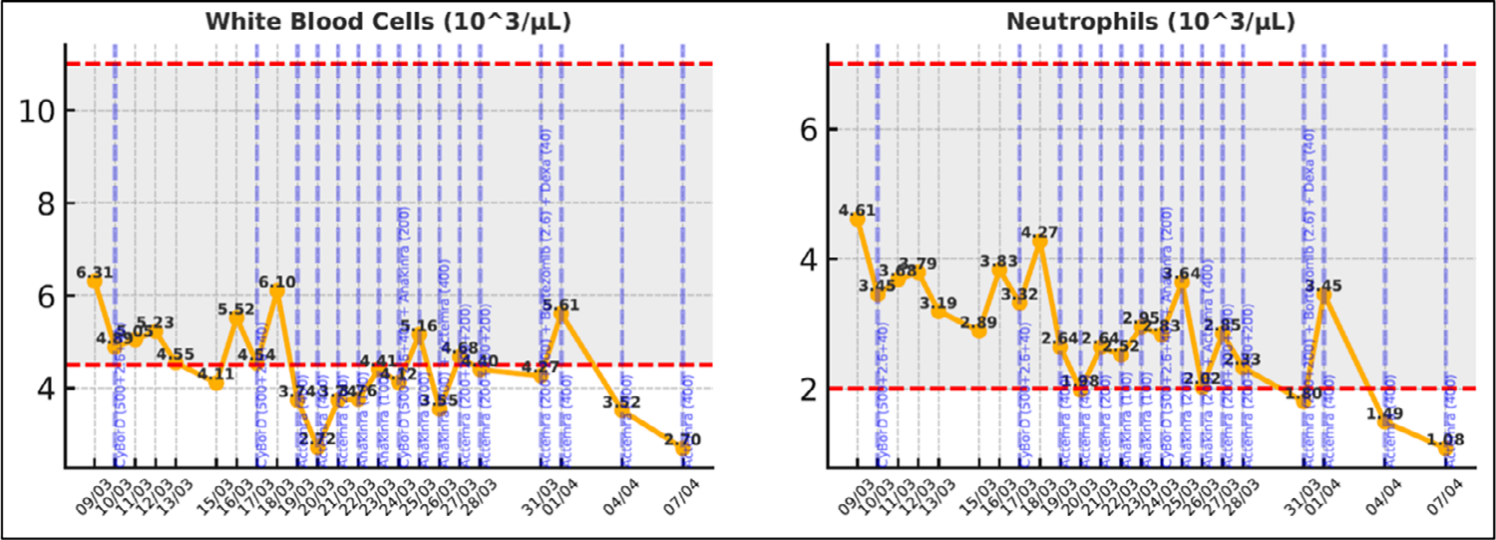
Evolution of white blood cells following initiation of treatment.

**FIGURE 3:**
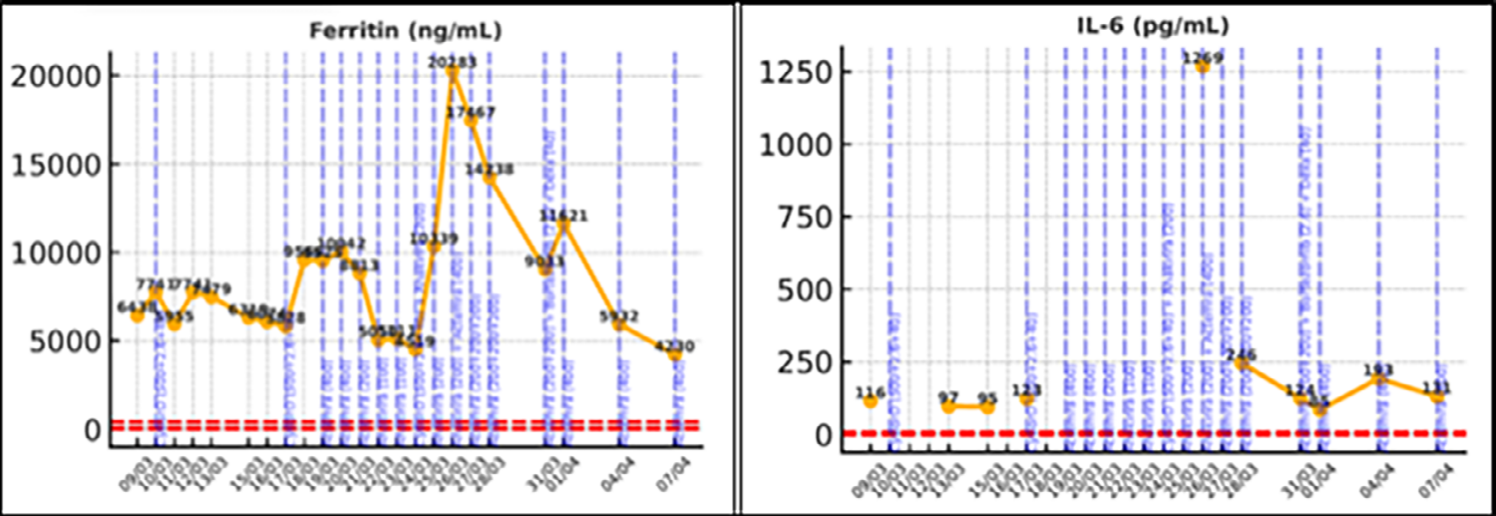
Evolution of Ferritin and IL‐6 following initiation of treatment.

Since the patient was unresponsive to high‐dose corticosteroids with poor symptom control, and met all nine of the nine diagnostic criteria included in the HScore, yielding a high pre‐test probability (96%–98%) for HLH, we recommended starting the patient on a regimen that would have activity on both myeloma and lymphoma clones: cyclophosphamide, bortezomib, and dexamethasone (CyBorD) and based on UK guidelines, recommended to start anakinra [11]. Despite these interventions, the inflammatory markers continued to rise with rapidly progressive clinical deterioration. Tocilizumab (Actemra) was initiated as salvage therapy with a response seen within 24 h (Figures 1, 2, 3). The patient was discharged home 10 days later, with an ongoing decrease in the inflammatory markers and improvement in the clinical symptoms (Supporting Information).

**FIGURE 4:**
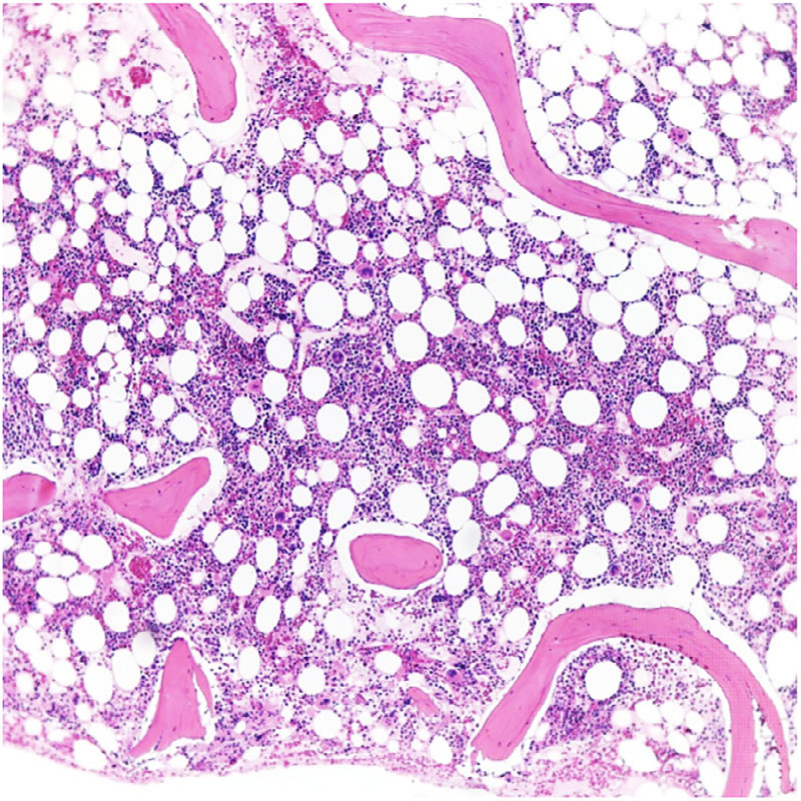
(Hematoxylin and Eosin, 5×). Hypercellular bone marrow for age with trilineage hematopoiesis.

**FIGURE 5:**
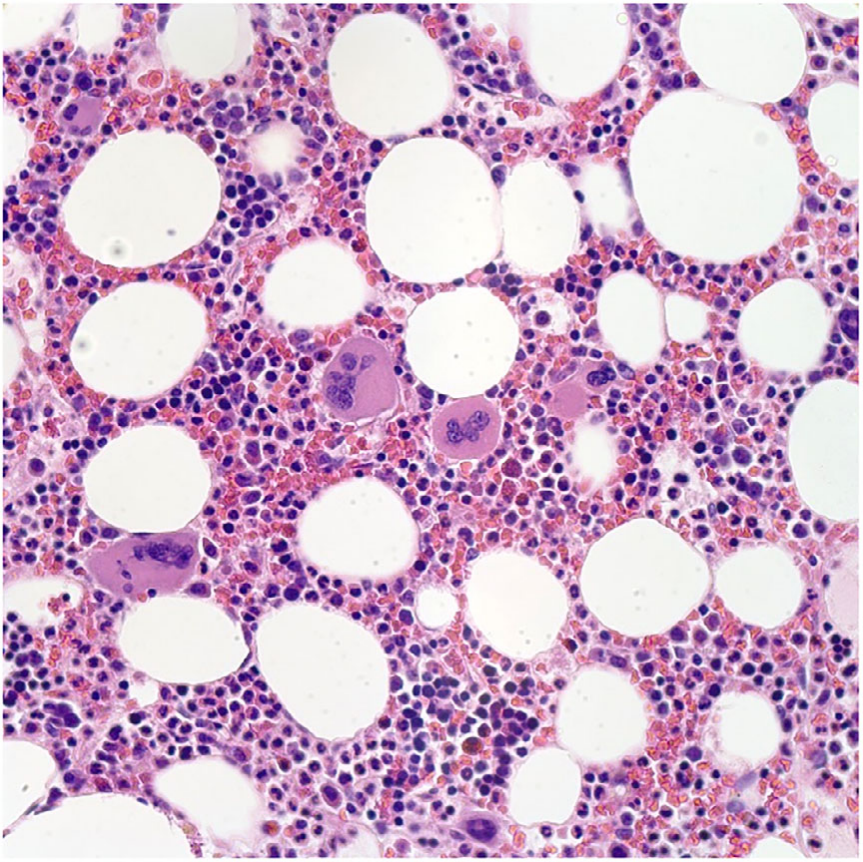
(Hematoxylin and Eosin, 40×). Bone marrow biopsy showing increased megakaryocytes.

**FIGURE 6:**
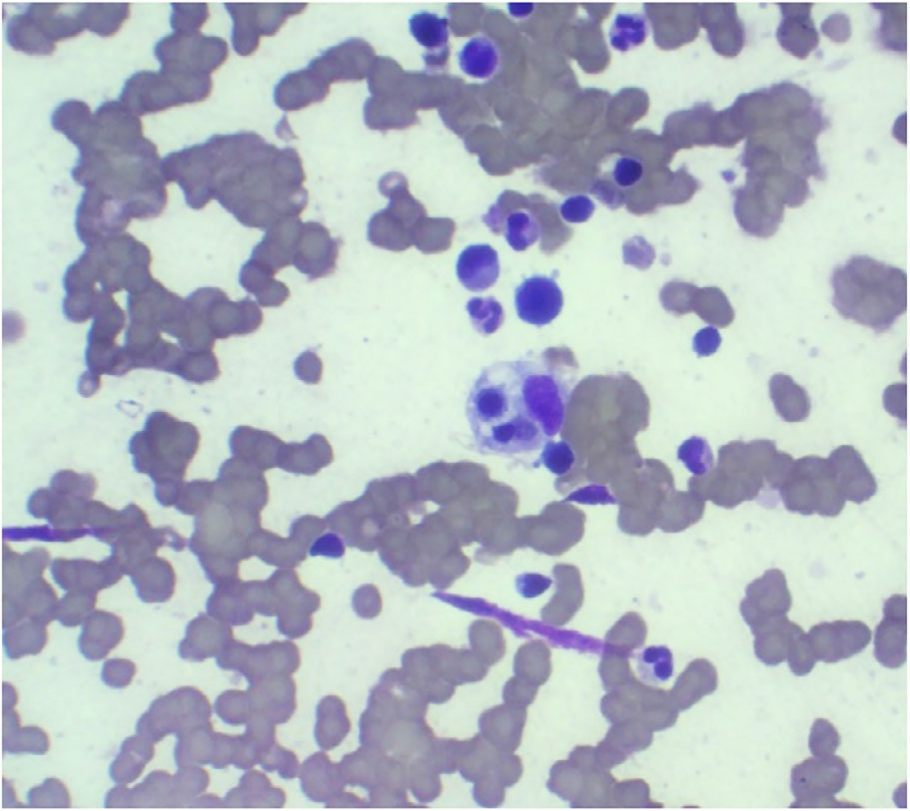
(Giemsa, 60×). Bone marrow aspirate smear showing hemophagocytosis (arrow).

**FIGURE 7:**
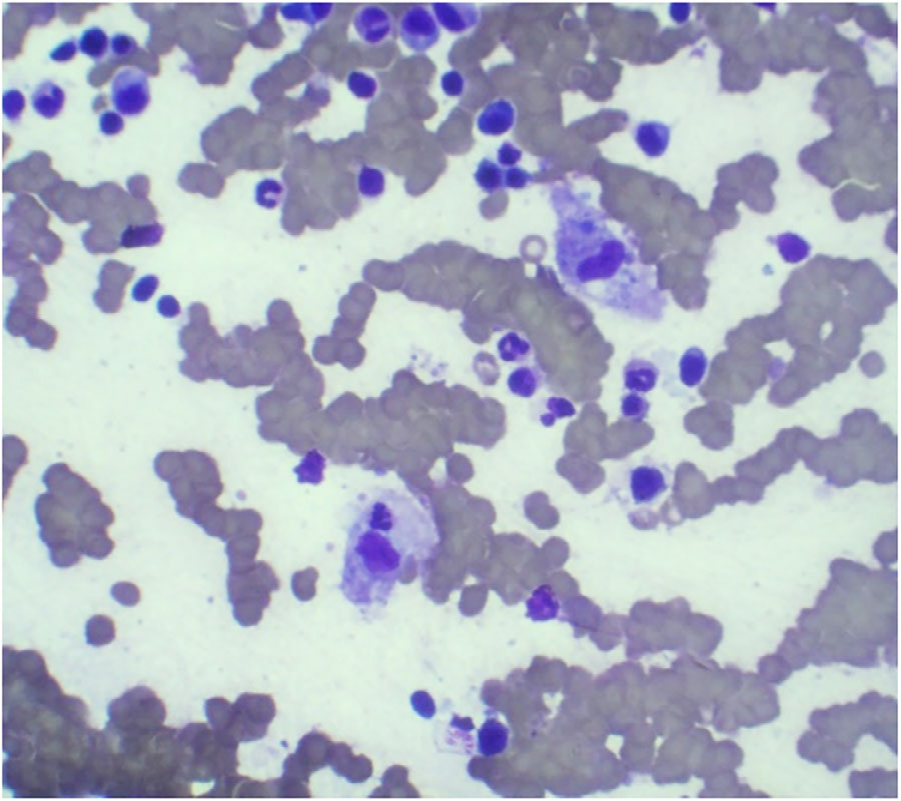
(Giemsa, 60×). Bone marrow aspirate smear showing hemophagocytosis of a neutrophilic precursor cell (arrow).

**FIGURE 8:**
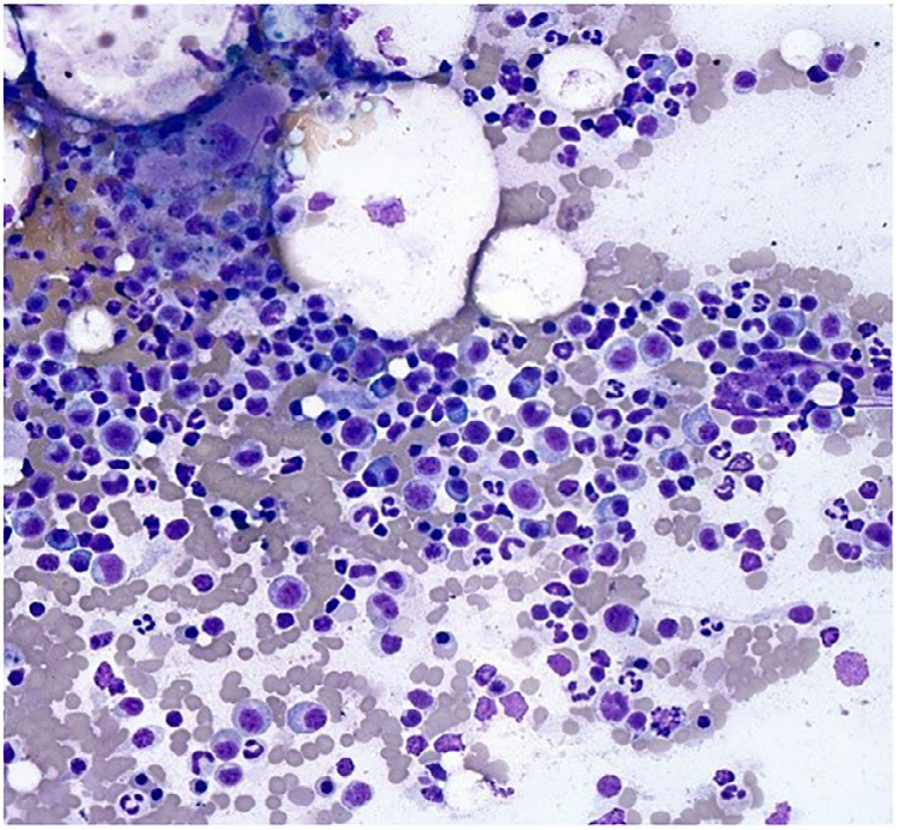
(Giemsa, 40×). Bone marrow aspirate smear showing increased plasma cells including binucleated forms.

**FIGURE 9:**
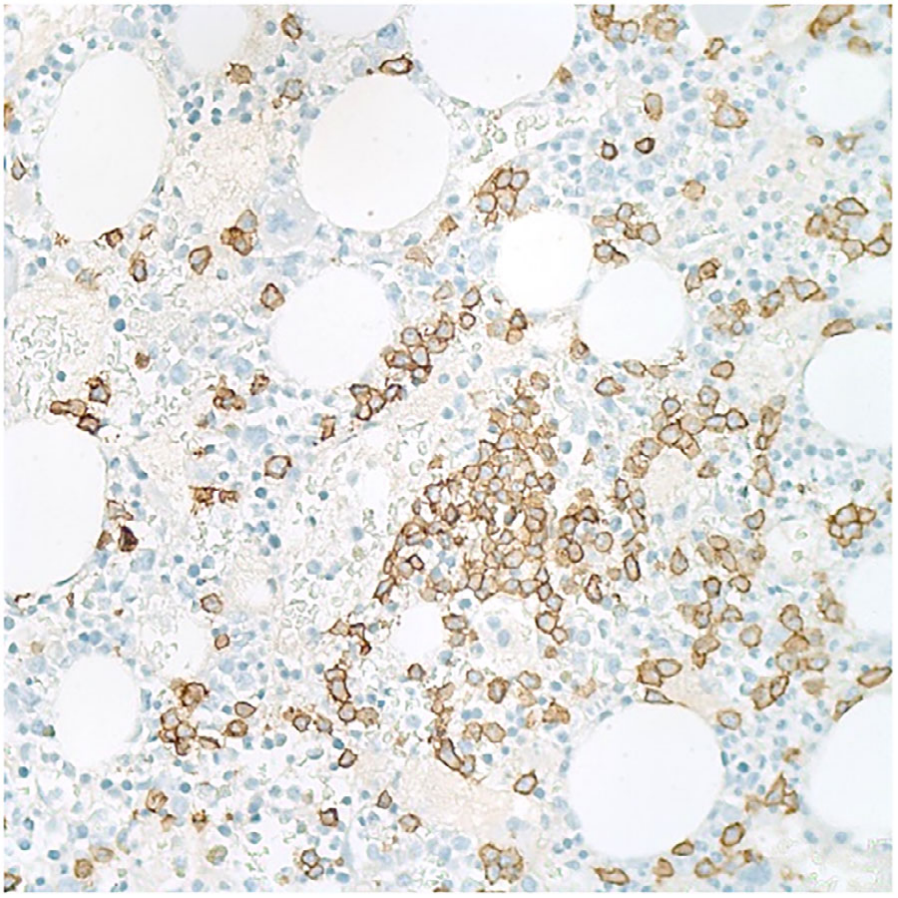
(CD138 IHC, 40×). Immunohistochemical stain for CD138 is positive highlighting focal increase in plasma cells, comprising 20%–30% of the marrow cellularity.

**FIGURE 10:**
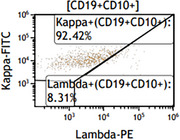
Flow cytometric analysis of the bone marrow aspirate showing a kappa‐restricted, CD10 positive monoclonal B‐cell population.

**FIGURE 11:**
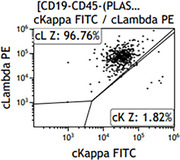
A lambda‐restricted, monoclonal plasma cell population was also identified with loss of CD19 expression.

## 3 Discussion

HLH associated with MM is a rare occurrence, particularly when it also involves an additional B‐cell malignancy. To our knowledge, this is the first report in the literature documenting HLH associated with two distinct untreated hematological malignancies.

One of the major challenges in treating patients with HLH is achieving a timely diagnosis. It is crucial to identify and address the underlying triggers of HLH. Despite increasing awareness among physicians and the rising incidence of HLH—especially with the growing use of immune‐activating and modulating agents—significant uncertainty still exists regarding the optimal diagnostic approach for this condition [4]. The patient met all the diagnostic criteria, which is uncommon. However, some results became available only after the therapy had been initiated, as time is of the essence when this diagnosis is high on the differential.

Various therapeutic strategies have been employed without clear management for someone with two different malignancies. Treatment typically involves a combination of HLH‐directed therapy and malignancy‐specific treatment [7]. The main goal of induction therapy is to suppress the inflammatory process. To achieve this, he was started on steroids and then followed up with chemotherapy, choosing a regimen that is effective for both myeloma and lymphoma. Anakinra, an IL‐1 antagonist that has been increasingly used in the treatment of secondary HLH, was also added [12]. Given the lack of initial response, and in coordination with the local team in Ecuador, we agreed to use tocilizumab while continuing chemotherapy. While emapalumab, an interferon‐gamma (IFN‐γ) blocking antibody, has been reported to have activity in HLH, this drug was not available in Ecuador. Tocilizumab is an anti‐IL‐6 receptor monoclonal antibody used for the management of cytokine release syndrome by inhibiting cytokines and signaling molecules involved in the inflammatory pathway [13]. While some prior reports cautioned against tocilizumab use due to limited efficacy [14, 15], treatment with tocilizumab resulted in a rapid improvement in symptomatology. Patient has started to wean off the drug and is on Cycle 2 of CyBorD.

This report highlights the rarity and complexity involved in diagnosing secondary HLH and its management. The case is significant not only for the rare diagnosis but also for the successful application of targeted therapy with tocilizumab to treat secondary malignancy‐associated HLH. This information may be of benefit to colleagues dealing with similar cases. We also would like to highlight the critical role that telemedicine played in the collaborative efforts surrounding the diagnosis and treatment of this patient, who was hospitalized abroad. Through secure and timely virtual consultations with the patient and local physician, telemedicine enabled minimizing geographical boundaries, by utilizing our collective expertise and developing an optimal care plan. This coordinated effort was instrumental in achieving the best possible outcome for the patient.

## Author Contributions

A.N., J.B., and L.S.C. were involved in patient care and collected clinical information. F.A. and V.S. did pathological analysis. C.R., A.L., and J.B. wrote the manuscript. All authors critically reviewed and approved the final manuscript.

## Funding

The authors have nothing to report.

## Ethics Statement

The authors have nothing to report.

## Consent

Consent was obtained from the patient for publication of this case report and any accompanying image.

## Conflicts of Interest

Jacqueline Barrientos has received speaker honoraria from Janssen, BeiGene, and AstraZeneca; is a consultant for BeiGene, AstraZeneca, Pharmacyclics, and Janssen; and has received research support from Merk, Nurix, and Abbvie. The other authors declare no conflicts of interest.

## Supporting information



Supporting Information

## Data Availability

All relevant data supporting the findings of this case report are included within the article. No additional datasets were generated or analyzed.
