# Supplementary material for: Hemophagocytic Lymphohistiocytosis as First Manifestation of Dual B‐Cell Neoplasms: A Case Report of Co‐Existing Multiple Myeloma and B‐Cell Lymphoma
Source: EJHaem. 2026 Jan 22;7(1):e70210. doi: 10.1002/jha2.70210 (PMC12826990; doi:10.1002/jha2.70210)
Supplement: Supplementary file 1 — Supporting Information [file JHA2-7-e70210-s001.docx]

| **Patient Perspective** |
| --- |
| My experience at Mount Sinai’s Comprehensive Cancer Center was both unique and profoundly impactful after enduring two months of persistent fever and undergoing numerous inconclusive tests. Within a week, I received a complex and rare diagnosis involving HLH (Hemophagocytic Lymphohistiocytosis), lymphoma, and myeloma. Although the diagnosis was difficult to process, the team acted swiftly and recommended immediate chemotherapy. They also initiated coordination with doctors in Ecuador to design a joint treatment plan, which included the Cybord protocol and consideration of an additional medication, Anakinra, known to be effective for HLH in other countries.  Upon returning to Ecuador, I began chemotherapy almost immediately. My condition was initially unstable, leading to hospitalization and a critical period during which an alternative medication, Actemra, was successfully administered. Actemra proved more effective in reducing inflammation, and I have continued with that treatment. Today, I feel significantly better, with all markers under control and no noticeable symptoms. Looking back, the care and support received—both at Mount Sinai and in Ecuador—were instrumental in helping me stay strong and hopeful. I remain deeply grateful for the expert coordination and the reassurance of being treated under the best hands possible to confront my difficult case and that have been a quite important factor to help me keep calm and confident that I will win this battle. |
